# Satisfaction survey among medical staff involved in relief operations following the Great East Japan Earthquake and Tsunami

**DOI:** 10.1186/cc11092

**Published:** 2012-03-20

**Authors:** Y Kondo, T Abe, S Deguchi, Y Kuba, H Mitsunaga, H Sekiguchi, K Kohshi, I Kukita

**Affiliations:** 1 University of the Ryukyus, Nishihara, Japan; 2 Mito Kyodo General Hospital, University of Tsukuba, Mito, Japan; 3 Graduate School of Health Sciences, Meio University, Nago, Japan; 4 Medical Corporation Kariyushi Heart-Life Hospital, Nishihara, Japan; 5 Tokyo Institute of Technology, Tokyo, Japan

## Introduction

We conducted an attitude survey regarding satisfaction among medical staff involved in relief operations following the Great East Japan Earthquake (magnitude 9.0) and Tsunami, which struck Japan on 11 March 2011. The damage was enormous and a number of medical relief teams visited the affected area to rescue victims. Our Okinawa medical relief team visited Otuchi, Iwate, on 15 March and provided medical support to the victims for 2.5 months.

## Methods

We conducted an anonymous paper survey using self-developed questionnaires. The 79 participants included medical doctors, nurses, and logisticians from medical relief teams involved in rescuing victims of the 2011 Great East Japan Earthquake and Tsunami. Data were analyzed using descriptive statistics. We also performed factor analysis to analyze responses with regard to factors such as face wash, toilets, sleep, clothes, and food.

## Results

The overall response rate was 59.5% (*n *= 47/79); the response rate was 38.3% (*n *= 18/47) for medical doctors, 36.2% (*n *= 17/47) for nurses, and 25.5% (*n *= 12/47) for logisticians. The mean length of career was 16.5 years (standard deviation, 9.75). Descriptive statistics revealed that the participants reported high satisfaction with regard to the command system and consistent satisfaction with regard to membership. However, some were unsatisfied with the deployment length. Almost all participants wanted to be part of a relief team if given an opportunity again. Factor analysis derived one factor (eigenvalue shows 3.48 (one factor), 0.33 (two factors), 0.17 (three factors), and 0.13 (four factors)) as comfort. Face wash (-0.95) contributed the most satisfaction compared to other factors such as toilets (-0.86), sleep (-0.81), clothes (-0.74), and food (-0.69).

## Conclusion

Almost all participants were satisfied with their level of comfort, and the influence of factors responsible for this comfort in descending order were face wash, toilets, sleep, clothes, and food.
